# Cognitive behavioral therapy and physical exercise for climacteric symptoms in breast cancer patients experiencing treatment-induced menopause: design of a multicenter trial

**DOI:** 10.1186/1472-6874-9-15

**Published:** 2009-06-06

**Authors:** Saskia FA Duijts, Hester SA Oldenburg, Marc van Beurden, Neil K Aaronson

**Affiliations:** 1 The Netherlands Cancer Institute, Division of Psychosocial Research and Epidemiology, Amsterdam, the Netherlands; 2 The Netherlands Cancer Institute, Department of Surgical Oncology, Amsterdam, the Netherlands; 3 The Netherlands Cancer Institute, Department of Gynecology, Amsterdam, the Netherlands

## Abstract

**Background:**

Premature menopause is a major concern of younger women undergoing adjuvant therapy for breast cancer. Hormone replacement therapy is contraindicated in women with a history of breast cancer. Non-hormonal medications show a range of bothersome side-effects. There is growing evidence that cognitive behavioral therapy (CBT) and physical exercise can have a positive impact on symptoms in naturally occurring menopause. The objective of this study is to investigate the efficacy of these interventions among women with breast cancer experiencing treatment-induced menopause.

**Methods/design:**

In a randomized, controlled, multicenter trial, we are evaluating the effectiveness of CBT/relaxation, of physical exercise and of these two program elements combined, in reducing menopausal symptoms, improving sexual functioning, reducing emotional distress, and in improving the health-related quality of life of younger breast cancer patients who experience treatment-induced menopause. 325 breast cancer patients (aged < 50) are being recruited from hospitals in the Amsterdam region, and randomly allocated to one of the three treatment groups or a 'waiting list' control group. Self-administered questionnaires are completed by the patients at baseline, and at 12 weeks (T1) and 6 months (T2) post-study entry. Upon completion of the study, women assigned to the control group will be given the choice of undergoing either the CBT or physical exercise program.

**Discussion:**

Cognitive behavioral therapy and physical exercise are potentially useful treatments among women with breast cancer undergoing treatment-induced, premature menopause. For these patients, hormonal and non-hormonal therapies are contraindicated or have a range of bothersome side-effects. Hence, research into these interventions is needed, before dissemination and implementation in the current health care system can take place.

**Trial registration:**

The study is registered at the Netherlands Trial Register (NTR1165) and ClinicalTrials.gov (NCT00582244).

## Background

Breast cancer is the most common cancer among women in the Netherlands, with an incidence of 71 per 100.000 person years, and a lifetime risk of 10%. [1] Nearly 25% of all women with breast cancer are premenopausal at the time of diagnosis. [2] Ovarian damage is the most significant long-term sequela of chemotherapy and/or endocrine treatment in premenopausal women. [3,4] It can lead to an earlier onset of menopause, with age and duration of treatment being its strongest predictors. [5] Premature menopause is a major concern of younger women undergoing adjuvant therapy for breast cancer [6], as effective and safe treatment options are not currently available.

### Premature menopause and menopausal symptoms

Primary menopausal symptoms include hot flushes, night sweats, vaginal dryness, decreased libido, dysuria and urinary incontinence. Secondary symptoms include insomnia due to night sweats, dyspareunia because of vaginal dryness, weight gain, and psychological distress. [3,7,8] Previous studies have indicated that breast cancer patients are more likely to experience menopausal symptoms than age-matched healthy controls, [9,10] and that especially younger breast cancer patients are affected by vasomotor and dystrophic symptoms. [7] Menopausal symptoms are an important source of morbidity [11] and discomfort [7,8] in patients with breast cancer and they may adversely affect women's sexual functioning, body image, and overall health-related quality of life. [6,9,12,13]

Among menopausal symptoms, hot flushes are considered to be the most disruptive, with prevalence rates between 63% and 80% in breast cancer patients. [8,14-17] Dysfunction of the thermoregulatory center in the hypothalamus due to (natural or treatment-induced) changes in estrogen levels has been postulated to be the cause of hot flushes. [18] As stress, spicy food and alcohol intake potentially affect the levels of serotonin, they may also increase the frequency and intensity of hot flushes. [19,20]

### Treatments for menopausal symptoms

Hormonal replacement therapy (HRT) is highly effective in alleviating vasomotor symptoms associated with menopause. [21] However, HRT is contraindicated in women with a history of breast cancer. [22] Some of the non-hormonal medications, e.g. clonidine and selective serotonin receptor inhibitors, have been shown to be moderately effective in reducing vasomotor symptoms associated with menopause. [23] However, they have a range of bothersome side-effects, such as nausea, dizziness and dry mouth. [24-27]

There is growing evidence that cognitive behavioral therapy and physical exercise can have a positive impact on symptoms in naturally occurring menopause. [28-30] Cognitive behavioral therapy (CBT) focuses on the relationships between thoughts, feelings and behavior, and is aimed primarily at stress reduction. CBT includes the following components: information about symptoms, monitoring and modifying precipitants, relaxation and stress management, cognitive restructuring of unhelpful assumptions and automatic thoughts, and encouraging helpful behavioral strategies. [28] A four session CBT including relaxation was found to be as beneficial in the reduction of vasomotor symptoms as HRT in a study of women undergoing natural menopause, and more effective than HRT in improving mood. [31]

Several studies have reported that relaxation therapy can effectively reduce hot flushes in naturally menopausal women, which may be explained by the reduction of sympathetic nervous system activity. [29,32,33] Significant associations have been observed between ratings of stress and reports of hot flushes, [19,20] suggesting that modification of precipitants of hot flushes and stress management may decrease the frequency and severity of hot flushes.

To our knowledge, only two studies have investigated the efficacy of behavioral interventions for the treatment of menopausal symptoms among women with breast cancer. In a randomized, prospective trial, Ganz et al. [34] found a significant, positive effect of a treatment package including counseling, pharmacological and behavioral interventions on menopausal symptoms and sexual functioning among women with a history of breast cancer. In a small randomised study (n = 16), relaxation training yielded a trend toward lower levels of hot flushes and night sweats, and a significant positive effect on psychological distress as compared to a control condition. [35]

A number of studies have generated results indicative of a beneficial effect of physical exercise on hot flushes occurring in naturally menopausal women. Two large population-based, cross-sectional studies carried out among Swedish women [36,37] indicated that regular vigorous exercise at least 3 hours per week is associated with a significantly lower risk of hot flushes. Only 5% of highly active women experienced severe hot flushes as compared to 15% of women who had little or no weekly exercise. It has been hypothesized that the positive effect of exercise on vasomotor symptoms may be due to elevated levels of endorphins that regulate central thermoregulation. [36,37] The findings of these observational investigations were further supported by two randomized studies conducted in Sweden and Japan [30,38] showing that a physical exercise program of at least 12 weeks had a significant positive effect on climacteric symptoms and health-related quality of life. However, one randomized study [39] has reported increased rates of hot flushes among obese, sedentary, postmenopausal women participating in an exercise program. This latter finding may be related to weight loss and decreased fat content leading to a decline in estrogen levels. [40]

Although cognitive behavioral therapy and physical exercise have been shown to have favorable effects in women undergoing natural menopause, these interventions have not yet been investigated in the context of menopausal symptoms experienced by breast cancer patients undergoing treatment-induced menopause.

### Current study

The present study is systematically evaluating the effectiveness of CBT/relaxation, of physical exercise and of these two program elements combined, in reducing menopausal symptoms, improving sexual functioning, reducing emotional distress, and in improving the health-related quality of life of younger breast cancer patients who experience treatment-induced menopause. In a randomized, controlled, multicenter trial, we are comparing CBT (A), physical exercise (B) and the combination of both interventions (AB) to a 'waiting list' control group.

We hypothesize that women in the CBT group, the physical exercise group or the combined group will report significantly greater reduction in overall levels of menopausal symptoms than patients in the usual care, waiting list control group. Furthermore, we hypothesize that women participating in the combined intervention will report significantly greater reduction in overall levels of menopausal symptoms than those participating in the CBT only or in the exercise only groups. Due to different mechanisms of action, we expect that the combined modalities will be more effective than either modality alone. CBT is expected to lead to significant reductions in menopausal symptoms via cognitive restructuring and stress management, while physical exercise is expected to impact primarily on metabolism of neurotransmitters responsible for thermoregulation. Finally, we hypothesize that women exposed to the interventions (groups A, B and AB) will report significantly more improvement in sexual functioning, body- and self-image, psychological distress, and generic health-related quality of life than those in the control group.

If demonstrated to be effective, the availability of such a structured supportive intervention program will be a welcome addition to standard medical treatment offered to breast cancer patients with treatment-induced menopause. It is anticipated that such a program will have direct benefit in terms of symptom relief and the improvement of patients' health related quality of life.

## Methods and design

**Figure 1:**
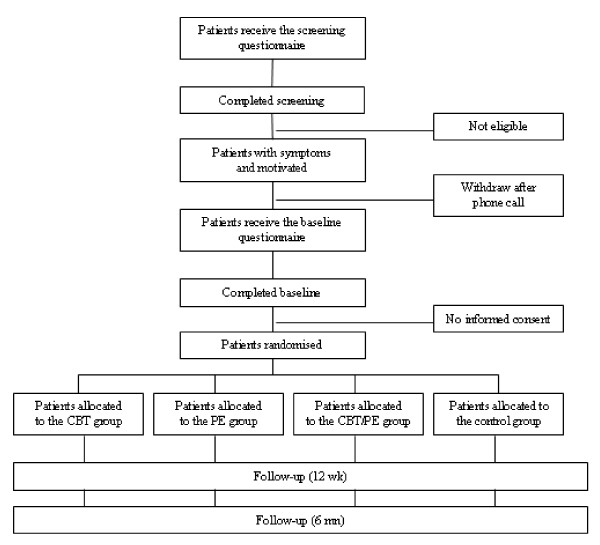
**Study flow diagram**.

Using a 2 × 2 full-factorial design patients are randomly allocated to one of the four following conditions: CBT, physical exercise, the combination of both interventions or a 'waiting list' control group. The design will allow for testing of the main effect of CBT, the main effect of physical exercise, and the additive or interactive effects of CBT and exercise on menopausal symptoms, the primary outcome measure. Secondary outcomes will include sexual functioning, urinary symptoms, body image and self-image, psychological distress and HRQL. The design of the study and the anticipated flow of the participants are graphically shown in Figure 1. The institutional review boards of all participating hospitals have approved the study. The study is registered at the Netherlands Trial Register (NTR1165) and ClinicalTrials.gov (NCT00582244).

### Study sample

The study sample will be composed of 325 women, younger than 50 years of age, with histologically confirmed primary breast cancer (stages: T1 – T4, N0 – N1 and M0). All women will have been premenopausal at the time of diagnosis, have completed adjuvant chemotherapy a minimum of 4 months and a maximum of 5 years prior to study entry, and will currently be disease-free. Hormonal therapy can still be ongoing. Potentially eligible women are screened for the presence of at least one of the following 3 menopausal symptoms during the previous week: hot flushes, sweating and/or vaginal dryness. Women are excluded from the study if they lack basic proficiency in Dutch, if they have serious cognitive or psychiatric problems, or serious physical comorbidity that would preclude them from participating in a physical exercise program. Since physical exercise may be contraindicated as a treatment for hot flushes in obese women, [39] patients with a BMI ? 30 are excluded from the study. Patients participating in concurrent studies or rehabilitation programs containing comparable psychosocial interventions are also excluded.

### Sample size

The FACT-ES scale, assessing endocrine symptoms [41] is the primary outcome on which sample size calculations are based. Specifically, mean group differences in the FACT-ES scores at 12 weeks and 6 months follow-up, controlling for baseline value, will serve as the primary endpoint of the study. With a total sample of 260 women (65 per group), and under the assumption of no interaction, the study will have 90% power to detect an effect size of 0.4 for the main effects of CBT (n = 130) and physical exercise (n = 130), with the p-value set at 0.05. If the combined effects of CBT and physical exercise are cumulative, or if the effect of CBT varies as a function of exposure to exercise or vice versa, (i.e. there is a significant interaction between CBT and exercise), this number of patients will allow testing each of the 4 cells in the factorial design (group A, B, AB and control group) separately with 80% power to detect an effect size of 0.5, again with p-value set at 0.05. [42]

We will recruit 325 women into the study, to allow for an attrition rate of approximately 20% (i.e. women who discontinue participation in the study entirely, including failure to complete all follow-up questionnaires; those women who discontinue participation in one of the groups but complete the follow-up assessments will be included in the analysis). Thus, 260 women will be available for the primary intention-to-treat analysis.

### Recruitment and randomisation

The patients are recruited from hospitals in the Amsterdam region. Potentially eligible patients are identified through hospital registries, for the 5-years retrospective inclusion, and through treating physicians, for the 2-years prospective inclusion. They are sent a letter signed by their physician informing them of the study and, if initially interested, asking them to complete a brief questionnaire to screen for menopausal symptoms. If they have no further interest in the study, they are asked to complete a postcard indicating reasons for not participating. In case of no response to the questionnaire or the postcard, a reminder is sent two weeks after the first mailing.

Those women who meet criteria for study entry based on their questionnaire responses are contacted by telephone to provide additional information on the study and to confirm their eligibility. In addition, they receive an extensive baseline questionnaire and informed consent form by mail. Computerized randomization takes place after response to both this baseline assessment and the informed consent. Block randomisation is used to ensure that each intervention consists of equally sized groups. In view of the nature of the interventions, blinding of the participants and the researchers is not possible.

During the follow-up period, self-administered questionnaires are sent to the patients at 12 weeks (T1) and 6 months (T2) post-study entry. Upon completion of the study, women assigned to the control group will be given the choice of undergoing either the CBT or physical exercise program.

### Interventions

The CBT and relaxation training consist of 6 weekly group sessions (with 6–8 participants per group) of approximately 1.5 hours duration. It also includes homework assignments (e.g. keeping a daily diary to monitor symptoms and their precipitants). The CBT program is based on the work of Hunter et al. [31] It comprises the following elements: (1) information and advice about symptoms (e.g. hot flushes, night sweats and sexual functioning); (2) monitoring and modifying precipitants; (3) relaxation and stress reduction; (4) cognitive restructuring of unhelpful thoughts, elicited by group discussion; and (5) encouraging helpful behavioral strategies (e.g. pacing activities). The primary focus of the CBT is on hot flushes and night sweats, but other symptoms (e.g. vaginal dryness) and problem areas such as sexuality, body- and self-image, and mood disturbance are also addressed. Relaxation is demonstrated and practiced during each session and an audio CD is provided for practicing at home. A 'booster' session is held approximately 6 weeks following completion of the initial 6 week program. The CBT is delivered by a social worker or psychologist and an assistant, usually an undergraduate psychology student.

The physical exercise program includes 4 individual contacts with a physiotherapist (one in-clinic intake of 90 minutes, two 15-minute telephone contacts, and a final 60-minute in-clinic session). Its core is an individually tailored, home-based and self-directed physical exercise program of 2.5–3 hours per week. During the first, supervised intake session, the exercise program is individually tailored, taking into consideration lifestyle, past and current levels of activity, preferences for types of activities (e.g. walking, cycling, gym exercises, etc.), and any disabilities. Each woman is provided with a heart-rate monitor, and is instructed in its use to achieve a target heart rate (60–80% Karvonen). In weeks 4 and 8, women have a brief telephone contact with the physiotherapist to discuss their experiences with the program and to make modifications, if necessary. Additionally, each week a telephone consultation hour is scheduled during which women can call the physiotherapist to discuss their exercise program, if desired. At the end of the program (week 12) women visit the clinic for a final session, during which advice is given on how best to maintain the level of physical activity (as used in the program) following completion of the program. At this time the data from the heart monitor are downloaded onto a computer.

Women assigned to the combined intervention group (AB) undergo the CBT and physical exercise elements of the program concurrently. To as great an extent as possible, the on-site CBT and in-clinic exercise training sessions are scheduled on the same day.

### Study measures

The patients' age, education, marital status, work status, weight and height, medication use (including alternative medications or therapies for menopausal complaints or depression) and life style variables (e.g. smoking, physical activity/exercise) are obtained via questionnaire. Clinical information, including date of diagnosis and tumor characteristics, and treatment history (type of surgery, possible breast reconstruction, radiotherapy, chemotherapy, endocrine treatments) are abstracted from the patients' medical records, if possible.

#### Overall levels of menopausal symptoms

The FACT-ES, an 18-item endocrine symptom scale, is used to assess overall levels of menopausal symptoms. [41] It includes both typical (vasomotor symptoms, vaginal dryness) and a-typical symptoms (e.g. weight gain, headaches) of menopause. The FACT-ES has been used among breast cancer patients treated by tamoxifen, [41] and in a sample of Dutch women at increased risk of breast/ovarian cancer who had undergone prophylactic oophorectomy. [43] The scale has shown high levels of reliability (Cronbach's alphas > 0.80 in both studies).

#### Vasomotor symptoms

Hot flushes and night sweats are assessed by the Hot Flush Rating Scale. [44] This 7-item questionnaire assesses the history of hot flushes, their current frequency, severity and duration (in the past week), emotional reactions, perceived disruptiveness, and perceived ability to cope with and control the symptoms. The scale has proven to have good test-retest reliability across a 2–3 week period (r = 0.80). The measure has been used among breast cancer patients treated with tamoxifen. [17]

#### Urinary symptoms

The Bristol Female Lower Urinary Tract Symptoms Questionnaire (BFLUTS) [45] assesses a wide range of urinary symptoms and their impact on sexual function and quality of life. It consists of 5 scales (34 items) assessing incontinence, voiding, filling, sexual function in relation to urinary problems and the impact on health related quality of life. For the purpose of the current study, only the 5-item incontinence scale will be used, including questions on urge, frequency, stress, unpredictable and nocturnal incontinence. The scale has shown to have good reliability (Cronbach's alpha = 0.75).

#### Sexuality

The Sexual Activity Questionnaire (SAQ) [46] is used to assess levels of sexual functioning. This 10-item measure consists of 3 scales: pleasure (desire, enjoyment, satisfaction and current frequency of activities); discomfort (vaginal dryness, pain and discomfort during penetration); and habit (frequency of sexual activity as compared to the usual level). The SAQ has been used in the British Tamoxifen Prevention Trial, [47] several studies of women with breast cancer, [48,49] and of women at increased risk of developing breast/ovarian cancer who had undergone prophylactic oophorectomy. [43] The SAQ has shown to have good reliability (Cronbach's alphas > 0.70).

#### Body image and self-image

Body image is assessed by the 4-item scale of the EORTC breast cancer-specific quality of life questionnaire, the QLQ-BR23. [50] This scale has been shown to have good reliability (Cronbach's alpha > 0.70) when employed in Dutch research settings. We have added two items to assess self-image, i.e. 'Have you been feeling older as a result of your treatment for breast cancer' and 'Did your self-image change as a result of your treatment for breast cancer?'

#### Psychological distress

Psychological distress is assessed with the 14-item Hospital Anxiety and Depression Scale (HADS). [51] The HADS assesses symptoms of mood disturbance, yielding separate scale scores for anxiety and depression, as well as a total score. Numerous studies have applied the HADS to assess distress among breast cancer patients. [52,53] The questionnaire has been validated for use in the Dutch population, [54] showing good psychometric properties (Cronbach's alphas of both the anxiety and depression scales > 0.79).

#### Generic health-related quality of life

The MOS SF-36 Item Health Survey (SF-36) [55] is used to assess generic health related quality of life. This 36-item questionnaire is organized into 8 multi-item scales assessing physical functioning, role functioning-physical, role functioning-emotional, pain, vitality, social functioning, mental health, and general health perceptions. Summary component scores for physical and mental health can also be calculated. It has been translated, validated and normed in a large number of languages, including Dutch. [56] It has also been used in previous studies of younger patients with breast cancer. [14] We have added one item to assess quality of sleep in the past month.

#### Compliance

Records are kept of the number of CBT sessions attended. Women are also asked to indicate the frequency and duration of relaxation sessions and other homework assignments. Records are also kept of attendance at the in-hospital exercise program sessions, and compliance with the home-based training is determined on the basis of both self-report and the data registered by the heart rate monitors. The monitors register frequency and duration of exercise, total exercise time in and outside the target zone, average heart rate of total exercise, and calorie expenditure.

Women who do not complete the entire program are asked to indicate the reason(s) for their discontinuation (e.g. illness, lack of motivation, burden, etc.). Every effort is made to obtain a final, post-intervention assessment for patients who discontinue the program. This facilitates a maximum sample size for the primary, intention-to-treat analysis. Additionally, all women, including those in the control group, are asked if, during the period of the study, they had pursued any (other) activities relating to their menopausal symptoms (e.g. alternative remedies).

#### Patients' evaluation of the intervention program

At the 12 week evaluation point, women in the 3 intervention arms of the study are asked to complete a brief series of questions about the perceived efficacy of and satisfaction with the intervention program, whether they would suggest any changes to the program, and if they would recommend it to other women experiencing symptoms of premature menopause.

### Statistical analyses

Analysis will first be performed to evaluate the comparability of the intervention and control groups at baseline in terms of sociodemographic and clinical characteristics. Student's t-test or appropriate non-parametric statistics will be used, depending on the level of measurement. If, despite the stratified randomization procedures, the groups are found not to be comparable on one or more background variables, those variables will be employed routinely as covariates in subsequent analyses.

Scores for the FACT-ES, the Hot Flush Rating Scale, the BFLUTS, the SAQ, the QLQ-BR23 body image scale, the HADS, and the SF-36 will be calculated according to published scoring algorithms. Between-group differences over time in mean scores will be tested using a two factor (group × time) multivariate analysis of variance with repeated measures on the time factor. Effect sizes will be calculated using standard statistical procedures. All analysis will, to as great an extent as possible, be conducted on an intention-to-treat basis. In addition, we will perform per-protocol analyses based on patients who complete the entire intervention. As indicated previously, the FACT-ES and the Hot Flush Rating Scale will be used as the primary study endpoints, and the remaining measures will be considered as secondary endpoints. For the analysis of these latter variables, appropriate statistical (p-value) adjustments will be made for multiple testing.

Supplementary analyses will be carried out in which data relating to compliance with the program elements is taken into account. Specifically, we will determine whether the level of compliance (based on attendance records, self-report data and, in the case of physical exercise, data stored in the heart rate monitor) is associated significantly with the changes over time in symptom relief, sexuality, body image, psychological distress, and health related quality of life.

## Discussion

Menopausal symptoms are common, and may be particularly severe in younger women with breast cancer who undergo treatment-induced menopause. Effective and safe treatment options for these symptoms in breast cancer patients are needed. Although CBT, relaxation and physical exercise have been found to have a positive effect on vasomotor symptoms in women undergoing natural menopause, they have not been investigated among women with breast cancer undergoing treatment-induced, premature menopause.

In the current study, we are systematically evaluating the effectiveness of CBT/relaxation, of physical exercise and of these two program elements combined, in reducing menopausal symptoms, improving sexual functioning, reducing emotional distress, and in improving the health-related quality of life of younger breast cancer patients who experience treatment-induced menopause.

### Methodological considerations

Our study has several strengths. First, the chosen research design – a 2 × 2 factorial design – will allow us to test the main effects of CBT and of physical exercise, and the additive or interactive effects of CBT and exercise. We believe that this is a robust and efficient design for testing these broad program elements. Second, the cognitive behavioral therapy under investigation was developed, and its effectiveness among natural menopausal women was evaluated by Hunter and colleagues. [31] Cognitive behavioral therapy resulted in significant reductions in hot flush frequencies by 59% (baseline vs. follow-up). Additionally, CBT also reduced anxiety and hot flush problem ratings. This study lends preliminary support to CBT as a potentially effective management approach for the reduction of hot flushes.

Several limitations of the study should be noted. First, a follow-up of 3 months is relatively short for an intervention study. However, it is similar to that used in previous studies on the effect of these interventions in women experiencing a natural menopause. Furthermore, all the outcomes at follow-up (with the exception of the data from the heart rate monitor) will be measured by self-report. Physiological assessment of hot flushes would provide more objective data, but is not feasible within the context of this multicenter study.

## Conclusion

Cognitive behavioral therapy and physical exercise are promising treatments among women with breast cancer undergoing treatment-induced, premature menopause. For these patients, hormonal and non-hormonal therapies are contraindicated or have a range of bothersome side-effects. Hence, research into these interventions is needed, before dissemination and implementation in the current health care system can take place. The current study will contribute to the knowledge on the effectiveness of cognitive behavioral therapy and physical exercise in younger breast cancer patients.

## Competing interests

The authors declare that they have no competing interests.

## Authors' contributions

NA, MvB and HO are the principal investigators of this study. SD is the postdoc on the study, and generated the first draft of this manuscript based on the study protocol. All authors approved the final version of the manuscript.

## Pre-publication history

The pre-publication history for this paper can be accessed here:



## References

[B1] Visser O, Siesling S, van Dijk J (2003). Incidence of cancer in the Netherlands 1999/2000: Eleventh report of the Netherlands Cancer Registry.

[B2] (2003). American Cancer Society – Cancer Facts Figures – 2000.

[B3] Bines J, Oleske DM, Cobleigh MA (1996). Ovarian function in premenopausal women treated with adjuvant chemotherapy for breast cancer. J Clin Oncol.

[B4] Shapiro CL, Recht A (2001). Side effects of adjuvant treatment of breast cancer. N Engl J Med.

[B5] Goodwin PJ, Ennis M, Pritchard KI, Trudeau M, Hood N (1999). Risk of menopause during the first year after breast cancer diagnosis. J Clin Oncol.

[B6] Avis NE, Crawford S, Manuel J (2004). Psychosocial problems among younger women with breast cancer. Psychooncology.

[B7] Biglia N, Cozzarella M, Cacciari F, Ponzone R, Roagna R, Maggiorotto F (2003). Menopause after breast cancer: a survey on breast cancer survivors. Maturitas.

[B8] Couzi RJ, Helzlsouer KJ, Fetting JH (1995). Prevalence of menopausal symptoms among women with a history of breast cancer and attitudes toward estrogen replacement therapy. J Clin Oncol.

[B9] Ganz PA, Rowland JH, Desmond K, Meyerowitz BE, Wyatt GE (1998). Life after breast cancer: understanding women's health-related quality of life and sexual functioning. J Clin Oncol.

[B10] Harris PF, Remington PL, Trentham-Dietz A, Allen CI, Newcomb PA (2002). Prevalence and treatment of menopausal symptoms among breast cancer survivors. J Pain Symptom Manage.

[B11] Angelopoulos N, Barbounis V, Livadas S, Kaltsas D, Tolis G (2004). Effects of estrogen deprivation due to breast cancer treatment. Endocr Relat Cancer.

[B12] Rostom AY (2001). The management of menopausal sequelae in patients with breast cancer. Clin Oncol (R Coll Radiol).

[B13] Young-McCaughan S (1996). Sexual functioning in women with breast cancer after treatment with adjuvant therapy. Cancer Nurs.

[B14] Bloom JR, Stewart SL, Chang S, Banks PJ (2004). Then and now: quality of life of young breast cancer survivors. Psychooncology.

[B15] Carpenter JS, Andrykowski MA (1999). Menopausal symptoms in breast cancer survivors. Oncol Nurs Forum.

[B16] Carpenter JS, Andrykowski MA, Cordova M, Cunningham L, Studts J, McGrath P (1998). Hot flashes in postmenopausal women treated for breast carcinoma: prevalence, severity, correlates, management, and relation to quality of life. Cancer.

[B17] Hunter MS, Grunfeld EA, Mittal S, Sikka P, Ramirez AJ, Fentiman I (2004). Menopausal symptoms in women with breast cancer: prevalence and treatment preferences. Psychooncology.

[B18] Shanafelt TD, Barton DL, Adjei AA, Loprinzi CL (2002). Pathophysiology and treatment of hot flashes. Mayo Clin Proc.

[B19] Gannon L, Hansel S, Goodwin J (1987). Correlates of menopausal hot flashes. J Behav Med.

[B20] Swartzman LC, Edelberg R, Kemmann E (1990). Impact of stress on objectively recorded menopausal hot flushes and on flush report bias. Health Psychol.

[B21] MacLennan A, Lester S, Moore V (2004). Oral oestrogen replacement therapy versus placebo for hot flushes (Cochrane Review).

[B22] Holmberg L, Anderson H (2004). HABITS (hormonal replacement therapy after breast cancer – is it safe?), a randomised comparison: trial stopped. Lancet.

[B23] Fugate SE, Church CO (2004). Nonestrogen treatment modalities for vasomotor symptoms associated with menopause. Ann Pharmacother.

[B24] Caley CF, Weber SS (1993). Paroxetine: a selective serotonin reuptake inhibiting antidepressant. Ann Pharmacother.

[B25] Kent JM (2000). SNaRIs, NaSSAs, and NaRIs: new agents for the treatment of depression. Lancet.

[B26] Laufer LR, Erlik Y, Meldrum DR, Judd HL (1982). Effect of clonidine on hot flashes in postmenopausal women. Obstet Gynecol.

[B27] Nagamani M, Kelver ME, Smith ER (1987). Treatment of menopausal hot flashes with transdermal administration of clonidine. Am J Obstet Gynecol.

[B28] Hunter MS (2003). Cognitive behavioural interventions for premenstrual and menopausal problems. J Reprod Infant Psychol.

[B29] Irvin JH, Domar AD, Clark C, Zuttermeister PC, Friedman R (1996). The effects of relaxation response training on menopausal symptoms. J Psychosom Obstet Gynaecol.

[B30] Lindh-Astrand L, Nedstrand E, Wyon Y, Hammar M (2004). Vasomotor symptoms and quality of life in previously sedentary postmenopausal women randomised to physical activity or estrogen therapy. Maturitas.

[B31] Hunter MS, Liao KLM (1996). Evaluation of a four session cognitive behavioural intervention for menopausal hot flushes. Br J Health Psychol.

[B32] Freedman RR, Woodward S (1992). Behavioral treatment of menopausal hot flushes: evaluation by ambulatory monitoring. Am J Obstet Gynecol.

[B33] Stevenson DW, Delprato DJ (1983). Multiple component self-control program for menopausal hot flashes. J Behav Ther Exp Psychiatry.

[B34] Ganz PA, Greendale GA, Petersen L, Zibecchi L, Kahn B, Belin TR (2000). Managing menopausal symptoms in breast cancer survivors: results of a randomized controlled trial. J Natl Cancer Inst.

[B35] Fenlon D (1999). Relaxation therapy as an intervention for hot flushes in women with breast cancer. Eur J Oncol Nurs.

[B36] Ivarsson T, Spetz AC, Hammar M (1998). Physical exercise and vasomotor symptoms in postmenopausal women. Maturitas.

[B37] Li C, Samsioe G, Borgfeldt C, Lidfeldt J, Agardh C, Nerbrand C (2003). Menopause-related symptoms: What are the background factors? A prospective population-based cohort study of Swedish women (The Women's Health in Lund Area study). Am J Obstet Gynecol.

[B38] Ueda M (2004). A 12-week structured education and exercise program improved climacteric symptoms in middle-aged women. J Physiol Anthropol Appl Human Sci.

[B39] Aiello EJ, Yasui Y, Tworoger SS, Ulrich CM, Irwin ML, Bowen D (2004). Effect of a yearlong, moderate-intensity exercise intervention on the occurrence and severity of menopause symptoms in postmenopausal women. Menopause.

[B40] Rice VM (2004). Effect of moderate-intensity exercise in alleviating menopausal symptoms. Menopause.

[B41] Fallowfield LJ, Leaity SK, Howell A, Benson S, Cella D (1999). Assessment of quality of life in women undergoing hormonal therapy for breast cancer: validation of an endocrine symptom subscale for the FACT-B. Breast Cancer Res Treat.

[B42] Cohen J (1988). Statistical power analysis for the behavioral sciences.

[B43] Madalinska JB, Hollenstein J, Bleiker EMA, van Beurden M, Valdimarsdottir HB, Massuger LF (2005). The quality of life effects of prophylactic salpingo-oophorectomy versus gynecologic screening among women at increased risk of hereditary ovarian cancer. J Clin Oncol.

[B44] Hunter MS, Liao KLM (1995). A psychological analysis of menopausal hot flushes. Br J Clin Psychol.

[B45] Brookes ST, Donovan JL, Wright M, Jackson S, Abrams P (2004). Then and now: quality of life of young breast cancer survivors. Am J Obstet Gynecol.

[B46] Thirlaway K, Fallowfield L, Cuzick J (1996). The Sexual Activity Questionnaire: a measure of women's sexual functioning. Qual Life Res.

[B47] Fallowfield L, Fleissig A, Edwards R, West A, Powles TJ, Howell A (2001). Tamoxifen for the prevention of breast cancer: psychosocial impact on women participating in two randomized controlled trials. J Clin Oncol.

[B48] Ganz PA, Desmond KA, Leedham B, Rowland JH, Meyerowitz BE, Belin TR (2002). Quality of life in long-term, disease-free survivors of breast cancer: a follow-up study. J Natl Cancer Inst.

[B49] Ganz PA, Greendale GA, Petersen L, Kahn B, Bower JE (2003). Breast cancer in younger women: reproductive and late health effects of treatment. J Clin Oncol.

[B50] Sprangers MA, Groenvold M, Arraras JI, Franklin J, te Velde A, Muller M (1996). The European Organization for Research and Treatment of Cancer breast cancer-specific quality-of-life questionnaire module: first results from a three-country field study. J Clin Oncol.

[B51] Zigmond AS, Snaith RP (1983). The hospital anxiety and depression scale. Acta Psychiatr Scand.

[B52] de Bock GH, Bonnema J, Zwaan RE, Velde CJ van de, Kievit J, Stiggelbout AM (2004). Patient's needs and preferences in routine follow-up after treatment for breast cancer. Br J Cancer.

[B53] Turner J, Kelly B, Swanson C, Allison R, Wetzig N (2005). Psychosocial impact of newly diagnosed advanced breast cancer. Psychooncology.

[B54] Spinhoven P, Ormel J, Sloekers PP, Kempen GI, Speckens AE, Van Hemert AM (1997). A validation study of the Hospital Anxiety and Depression Scale (HADS) in different groups of Dutch subjects. Psychol Med.

[B55] Ware JEJ, Sherbourne CD (1992). The MOS 36-item short-form health survey (SF-36). I. Conceptual framework and item selection. Med Care.

[B56] Aaronson NK, Muller M, Cohen PD, Essink-Bot ML, Fekkes M, Sanderman R (1998). Translation, validation, and norming of the Dutch language version of the SF-36 Health Survey in community and chronic disease populations. J Clin Epidemiol.

